# Evaluation of CD1a immunostaining in the diagnosis of cutaneous leishmaniasis caused by *Leishmania donovani* in Sri Lanka

**DOI:** 10.1017/S0031182024000799

**Published:** 2024-09

**Authors:** Hasna Riyal, Nilakshi Samaranayake, Priyani Amarathunga, Deepani Munidasa, Nadira Karunaweera

**Affiliations:** 1 Department of Parasitology, Faculty of Medicine, University of Colombo, Colombo, Sri Lanka; 2 Department of Pathology, Faculty of Medicine, University of Colombo, Colombo, Sri Lanka; 3 Teaching Hospital, Anuradhapura, Sri Lanka

**Keywords:** biopsy, CD1a, cutaneous leishmaniasis, immunodiagnostics, immunohistochemistry, *Leishmania donovani*

## Abstract

Cutaneous leishmaniasis (CL) is a vector-borne parasitic disease, routinely diagnosed by direct light microscopy. The sensitivity of this method is dependent on the number of parasites present in the lesion. Immunoexpression of CD1a surface antigen by *Leishmania* amastigotes and its application as a diagnostic tool has been recently demonstrated in several species including *Leishmania major*, *Leishmania tropica* and *Leishmania infantum*. *Leishmania donovani* is the only reported species in Sri Lanka primarily causing CL and its CD1a status remains unexplored. We studied CD1a expression by amastigotes of *L. donovani* in skin biopsies from 116 patients with suspected CL. The biopsy sections were stained with CD1a clones O10 and MTB1 separately. Slit skin smear (SSS) results were considered the gold standard for diagnosis of CL. 103 cases were confirmed through SSS where 73 of them showed positive parasite staining for CD1a clone MTB1 with 70.9% sensitivity. Positivity was seen mostly in parasites closer to the epidermis. CD1a clone O10 failed to detect any amastigotes. Test sensitivity improved to 74.1% when the analysis was applied only to patients with low/no discernible Leishman-Donovan (LD) bodies in histology. Our findings show that CD1a clone MTB1 successfully stains amastigotes of *L. donovani* species and can be used as a supplementary diagnostic tool in detecting CL, especially when LD bodies are low in number. This method could be validated to detect other forms of leishmaniasis caused by *L. donovani* in Indian and sub-Saharan regions.

## Graphical abstract

**Figure:**
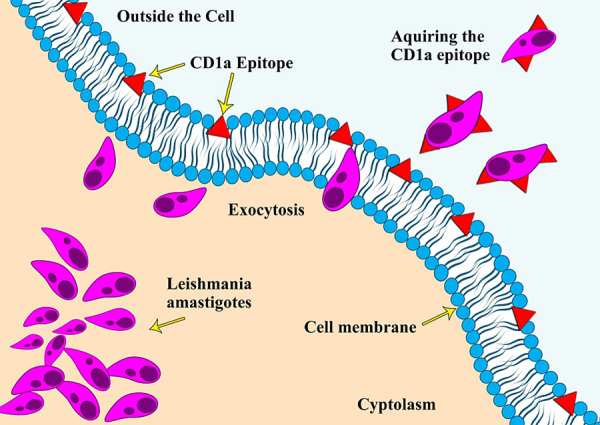

## Introduction

Leishmaniasis is a parasitic disease caused by protozoans of the genus *Leishmania* and constitutes a significant global health concern. The disease manifests in three main forms: cutaneous leishmaniasis (CL), mucocutaneous leishmaniasis (MCL) and visceral leishmaniasis (VL). In some regions, there is a non-lethal complication of VL that emerges months or even years after completing treatment for VL, known as post-kala-azar dermal leishmaniasis (PKDL) (Chapman *et al*., 2020). CL is the commonest form of disease with 600 000 to 1 million new cases occurring annually (WHO, 2023).

The gold standard for diagnosing CL is the identification of parasites, primarily based on the detection of amastigotes in lesion material through microscopic examination. However, this diagnostic method relies heavily on the abundance of parasites present. Other diagnostic techniques include histological investigations to visualize LD bodies, culturing of parasites and PCR. Nevertheless, these latter two methods are not routinely available in most hospital settings in countries such as Sri Lanka due to cost and the requirement for skilled personnel. Additionally, there are instances where patients do not exhibit positivity by any of the above methods, making it challenging for dermatologists to confirm the diagnosis, especially when the clinical presentation is less suggestive of CL. Even though a Rapid Diagnostic Test (RDT) is available for the detection of CL, it has proven to be of low sensitivity in many settings including Sri Lanka (Gebremeskele *et al*., 2023; Piyasiri *et al*., 2023). Limiting the burden of CL depends on strengthening both the diagnostic and therapeutic aspects. While new treatment methods are being explored to overcome the drawbacks of conventional chemotherapy (Dar *et al*., 2020; Iqbal *et al*., 2022; Khalid *et al*., 2022), it is timely to also explore complementary methods that would add to the diagnostic repertoire.

CD1a is a surface protein expressed on Langerhans cells and dendritic cells and is often used in immunohistochemistry to visualize the presence of these immune cell types (Dougan *et al*., 2007). An unusual phenomenon of *Leishmania* amastigotes expressing the CD1a molecule and the possibility of using it as an immunodiagnostic tool was reported for the first time in 2012, during an immunohistochemical (IHC) expression study on leishmaniasis caused by *L. major* and *L. tropica* (Karram *et al*., 2012). Researchers have proposed various theories regarding the acquisition of this CD1a host molecule by the parasites, with one of the most reported theories being that it gets attached during the exocytosis process of the parasite from the host cells (Karram *et al*., 2012). Some scientists also argue that it could be due to the cross-reactivity of antibodies with the parasite's glycocalyx. Notably, the absence of the CD1a molecule in promastigote cultures indicates that the acquisition of this molecule likely occurs only during host infectivity (Jabbour *et al*., 2015). Since the initial report, only a few studies have explored this phenomenon of immune mimicry in other *Leishmania* species (Fernandez-Flores and Rodriguez-Peralto, 2016; Dias-Polak *et al*., 2017; Sundharkrishnan and North, 2017; DeCoste *et al*., 2020; Karabulut *et al*., 2021). These studies suggest that not all *Leishmania* species are positive for CD1a, particularly those responsible for New World leishmaniasis (Sundharkrishnan and North, 2017; Ferrufino-Schmidt *et al*., 2019).

The Indian sub-continent (India, Pakistan, Sri Lanka, Nepal, Bangladesh & Bhutan) primarily reports VL caused by *L donovani,* where the CD1a status of the parasite is not known. In Sri Lanka, molecular evidence has confirmed the causative agent of both CL and VL to be *L. donovani* (Karunaweera *et al*., 2003; Samarasinghe *et al*., 2018) with CL comprising almost the total case burden. Therefore, in this study, we utilized biopsy specimens from patients with locally acquired disease, both CL positive and negative, and stained their histology sections with CD1a clones O10 and MTB1 to assess the CD1a status of the parasite species *L. donovani*.

## Materials and methods

A total of 103 patients with a confirmed diagnosis of CL through slit skin smear (SSS), attending the Dermatology Clinic at Teaching Hospital, Anuradhapura, North-Central Province of Sri Lanka, between January 2018 and November 2018, were enrolled in the study. The sample size was calculated using the *N* = z^2^(pq)/d^2^ formula where *P* was 45% according to a similar marker positivity observed in a Sri Lankan CL study previously (Lwanga *et al*., 1991; Ranawaka *et al*., 2012). Participants were between 18 and 70 in age and informed written consent was obtained from all the participants.

A 3 mm punch biopsy was obtained from the edge of each lesion. The biopsy was then gently rolled over a glass microscope slide to create an impression smear to aid in the CL diagnosis. Subsequently, the biopsy specimens were processed into Formalin-Fixed, Paraffin-Embedded (FFPE) tissue blocks. For histopathological evaluation, 5 *μ*m thick sections were cut and stained with Hematoxylin and Eosin (H&E). The parasite load (LD bodies) was assessed based on a modified Ridley's classification, with a score of 0 indicating no parasites, +1 for 1–10 parasites, +2 for 10–100 parasites, and +3 for 100 or more parasites per standard section (Ridley and Ridley, 1983). A parasitic index of 0 was defined as ‘low’, +1 and +2 were defined as ‘moderate’ and a parasitic index of +3 was defined as ‘high’.

Additional 4*μ*m thick sections were obtained from the tissue blocks for immunohistochemical staining with the two distinct anti-CD1a antibody clones on separate slides. After heat-induced antigen retrieval, immunohistochemical reactions were performed using anti-CD1a antibody clone MTB1 (Invitrogen/ThermoFisher Scientific, USA Cat#MA5-14096, concentrated) at a 1:15 dilution, and clone O10 (DAKO/Agilent Technologies Inc., USA, predilute). The incubations were carried out for one hour at room temperature, and visualized using EnVision FlexTM polymer (DAKO/Agilent Technologies Inc., USA). All reactions were manually performed with appropriate histochemistry controls such as antibody control, positive tissue control, negative tissue control and isotype control.

Thirteen patients who tested negative for CL through SSS, biopsy impression smear and histology were also included in the study as a CL-negative group for evaluation of the immunostaining. IHC staining with both CD1a clones were done on the CL negative group as well. The pathologist reviewing the IHC slides was blinded to SSS results and the IHC slides were reviewed independently of the H&E slides.

### Statistical analysis

Clinicopathological features were assessed against CD1a staining outcome using Chi-square statistics. Differences were considered significant when *P* values were *<0.05*. To evaluate the diagnostic performance of the immunohistochemical staining for CD1a, sensitivity, specificity, accuracy, positive predictive value and negative predictive value were calculated, with the SSS results as the gold standard (Baratloo *et al*., 2015; Safari *et al*., 2015). *K*-value was calculated to assess the agreement between the two tests. All data were analysed using IBM SPSS statistics version 23 software (SPSS Inc., Chicago, IL, 60606, USA).

## Results

**Figure 1.**
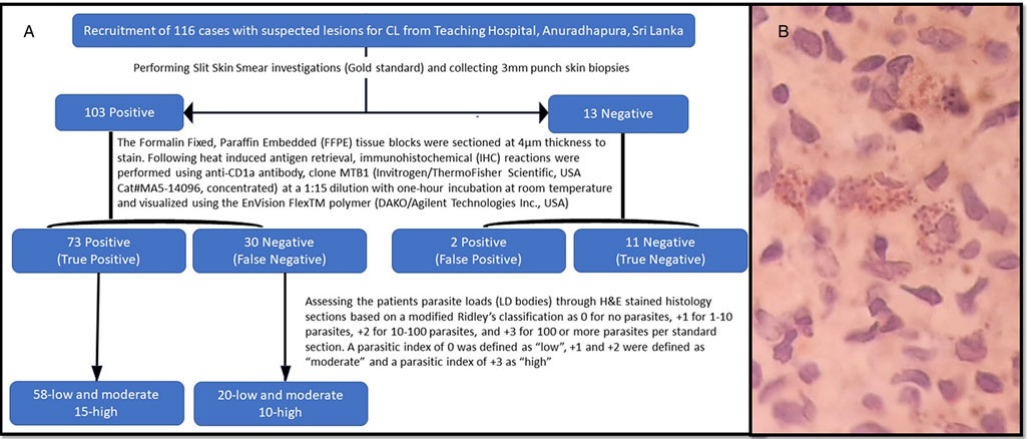
The workflow used to detect the positive staining of amastigotes using CD1a clone MTB1: (a) Procedure for participant recruitment and testing for amastigote visualization. (b) An enlarged image of *L. donovani* amastigotes successfully stained with CD1a clone MTB1 (x1000). The staining was done in parallel with IHC controls as follows: Positive tissue control – A well-known positive CL section with a large number of visible amastigotes; Negative control – A section that was triple negative for CL diagnosis (SSS, impression smear, and H&E); Antibody control – A well-known positive CL section stained without the primary antibody (replaced with distilled water); Isotype control – A well-known positive CL section stained with CD1a clone O10.

The majority of the patients were males (*n* = 77, 74.8%) and the patient's age ranged from 18 to 70 years with a mean age of 41.66 ± 2.509 (CI 95%) (Table 1). The detailed sociodemographic features of the patients have been reported previously (Riyal *et al*., 2023). Out of the 103 patients included in the study, 34 showed LD bodies in the histology sections, where 25 of them were classified as having a high parasite load, as described in the Methods section. CD1a clone MTB1 displayed positive parasite staining in 75 cases (Fig. 1), two of which were false positives according to SSS results.

**Table 1.** Demographics and clinicopathological features of the patients

Basic demographics of the patients		
Gender		
Male		77 (74.8%)
Female		26 (25.2%)
Age distribution		
11–25		9 (8.7%)
26–40		45 (43.7%)
41–55		31 (30.1%)
56–70		18 (17.5%)
Clinicopathological feature	Patients positive for CD1a clone MTB1 *n* (%)	Patients negative for CD1a clone MTB1 *n* (%)
Granuloma formation		
Poor-formed granuloma	43 (58.9%)	18 (60%)
Well-formed granuloma	30 (41.1%)	12 (40%)
Intensity of the inflammation		
Superficial inflammation	25 (34.2%)	15 (50%)
Whole dermis inflammation	48 (65.8%)	15 (50%)
Lesion type		
Papules & nodules	45 (61.6%)	14 (46.7%)
Ulcers & scars	28 (38.4%)	16 (53.3%)
Duration of the lesion		
Less than 3 months	41 (56.2%)	15 (50%)
More than 3 months	32 (43.8%)	15 (50%)

**Figure 2.**
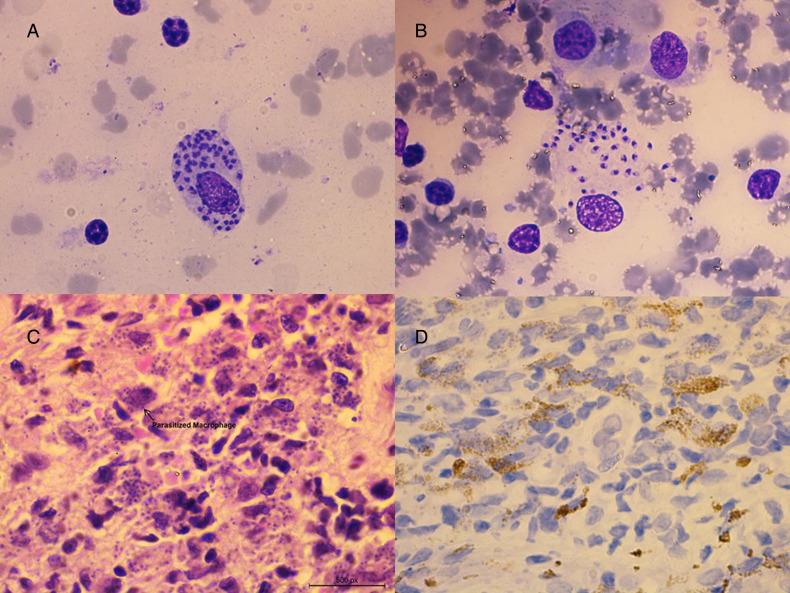
Four detection methods for identifying amastigotes using cytological and histological specimens. (a) a parasitized macrophage with numerous amastigotes detected in a Geimsa-stained slit skin smear (x1000) (b) numerous amastigotes detected in Geimsa-stained biopsy impression smear (x1000) (c) numerous LD bodies detected in an H&E-stained histology section (x400) (d) numerous LD bodies stained with CD1a clone MTB1 in an IHC section (x400).

The parasite staining with CD1a clone MTB1 was predominantly observed in the upper dermis with variable intensity. Detailed characteristics were not easily distinguishable; however, staining was accentuated on one side of the structure corresponding to the kinetoplast of the parasite (Fig. 1). Figure 2 illustrates the four different methods adopted for diagnosing CL using lesion materials.

**Figure 3.**
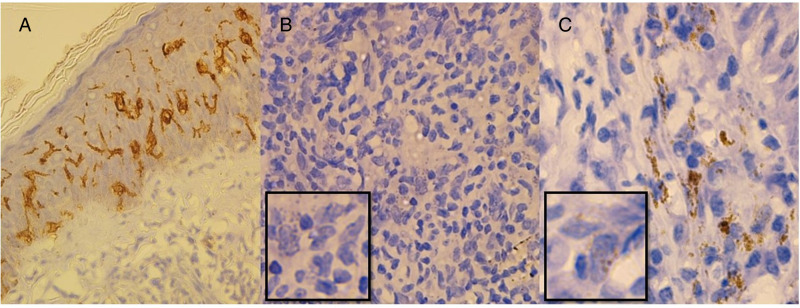
CD1a staining patterns in CL patients. (a) Histology positive for CL stained with CD1a clone O10 showing mostly Langerhans cells (x100) (b) Histology positive for CL with high LD body numbers, but none of the parasites are stained with CD1a clone MTB1 (x200) (c) Histology positive for CL with fewer LD body numbers stained with CD1a clone MTB1 (x400).

In contrast, immunostaining to visualize parasites with CD1a clone O10 was not successful, as it predominantly showed immune cells positive for the marker, particularly Langerhans cells found near the basal layer (Fig. 3a). Moreover, the number of parasites showing positive staining for clone MTB1 decreased gradually through the depth of the dermis. Notably, sections with high parasite loads had a low probability of being stained with clone MTB1, leading to a significant number of false-negative cases (Fig. 3b). Among the 25 cases with high parasite loads detected through H&E, only 15 (15/25, 60%) showed CD1a positivity for amastigotes through IHC. Chi-square statistics indicated that none of the clinicopathological features influenced the outcome of amastigotes getting stained with CD1a clone MTB1 (Table 1).

The test statistics for the detection of parasites through CD1a clone MTB1 are summarized in Table 2. To determine if this diagnostic method is more suitable for patients with low or no LD bodies, test statistics were recalculated on the same cohort after excluding the 25 patients with high parasite loads (>100 LD bodies). As a result, the sensitivity of the test increased from 70.9 to 74.4% (Table 2). Likewise, other test statistics also improved when analysing the reduced patient subset (Table 2).

**Table 2.** Comparison of diagnostic performance of CD1a clone MTB1 immunostaining against slit skin smear results

CD1a clone MTB1 staining	Microscopic results for SSS (gold standard)	
	Positive (103)	Negative (13)
Test positive (75)	True positive (73)	False positive (2)
Test negative (41)	False negative (30)	True negative (11)
A		
Accuracy = 72.4%		
Sensitivity = 70.9%		
Specificity = 84.6%		
Positive predictive value = 97.3%		
Negative predictive value = 26.8%		
*K*-value = 0.26		
CD1a clone MTB1 staining	Microscopic results for SSS (gold standard)	
	Positive (78)	Negative (13)
Test positive (60)	True positive (58)	False positive (2)
Test negative (31)	False negative (20)	True negative (11)
B		
Accuracy = 75.8%		
Sensitivity = 74.4%		
Specificity = 84.6%		
Positive predictive value = 96.7%		
Negative predictive value = 35.4%		
*K*-value = 0.37		

A-CD1a clone MTB1 staining results against all the slit skin smear results (*n* = 116)

B-CD1a clone MTB1 staining results against the slit skin smear results (patients with >100 LD bodies seen in histology are excluded) (*n* = 91).

## Discussion

To the best of our knowledge, this study is the first to assess the immunodiagnostic potential of the CD1a marker for diagnosing CL caused by *L. donovani*. A previous study reported that Old World *Leishmania* species exhibited 71% positivity for this marker compared to New World *Leishmania* species, which showed only 44% positivity (Sundharkrishnan and North, 2017). Our findings align with these results, as *L. donovani*, an Old World *Leishmania* species (Kevric *et al*., 2015), in this instance has shown a positivity rate of 70.9%.

The differential performance of various CD1a clones in detecting amastigotes is a noteworthy observation. Studies have highlighted the clone specificity of the antibody involved in this phenomenon, with CD1a clone O10 showing poor staining of amastigotes (McCalmont, 2012). Conversely, another CD1a clone, EP3622, had successfully stained amastigotes of *L. infantum* in previous studies with sensitivity ranging from 71.4 to 94% (DeCoste *et al*., 2020; Karabulut *et al*., 2021; Lopez-Trujillo *et al*., 2021). The difference in species as well as the number of samples tested, are other likely factors contributing to the differences in the reported sensitivity of the assay.

The localization of amastigote staining by CD1a predominantly in the papillary dermis, with lower expression towards the deep dermis, especially in cases of high parasite loads, is consistent with previous findings by Fernandez-Flores and Rodriguez-Peralto (Fernandez-Flores and Rodriguez-Peralto, 2016). These authors hypothesized that amastigotes that escape to the deep dermis without undergoing an exocytosis process by a host cell may give rise to CD1a-negative amastigotes through binary fission. Furthermore, they demonstrated that acquiring CD1a at deeper levels is nearly non-existent, as amastigotes have no source of Langerhans cells at the deep dermis (Fernandez-Flores and Rodriguez-Peralto, 2016). Langerhans cell populations getting depleted over the chronicity of the infection due to exhaustion of antigen presentation could also be a reason for not having CD1a-stained amastigotes in patients with high parasite loads (Meymandi *et al*., 2004).

The significance of our study lies in its relatively high sensitivity (>70%) for diagnosing *L. donovani*, particularly in cases with no or low discernible LD bodies in histology. This contrasts with the reported sensitivity levels for other *Leishmania* species, of 24.3% for *L. major* and *L. tropica* causing CL (Dias-Polak *et al*., 2017) and 58.3% for *L. infantum* causing VL (Gadelha *et al*., 2019). To enhance the reliability of test specificity values, we recommend the inclusion of a larger CL-negative patient cohort. Adding PCR as a reference test to check the performance of the index test holds significance as it can reveal the parasite species as well. However, PCR is not routinely performed in our setting for the diagnosis of CL due to the high cost. Additionally, we emphasize the importance of replicating this experiment in studies investigating all clinical forms of leishmaniasis caused by *L. donovani*, including VL and PKDL, to evaluate the significance of CD1a clone MTB1 as a diagnostic marker of disease caused by this species. The performance of this test suggests the potential for it to be developed as a reliable second-line investigation, on occasions a skin biopsy is performed due to doubtful clinical diagnosis.

In conclusion, our study demonstrates that CD1a clone MTB1 successfully stains amastigotes of *L. donovani*, making it a promising supplementary diagnostic tool for detecting CL, especially in cases where LD bodies are scarce. These findings have significant implications for improving the accuracy of CL diagnosis and may contribute to the development of more effective diagnostic strategies in the future.

## Data Availability

The data that support the findings of this study are available on request from the corresponding author.
